# Very Early-Life Risk Factors for Developing Dementia: Evidence From Full Population Registers

**DOI:** 10.1093/geronb/gbad142

**Published:** 2023-09-26

**Authors:** Martin Fischer, Martin Lövdén, Therese Nilsson, Dominika Seblova

**Affiliations:** Department of Clinical Sciences/Faculty of Medicine, Lund University, Malmö, Skåne County, Sweden; RWI—Leibniz Institute for Economic Research, Essen, Germany; Department of Psychology, University of Gothenburg, Gothenburg, Västra Götaland County, Sweden; Department of Economics, Lund University, Lund, Skåne County, Sweden; Research Institute of Industrial Economics (IFN), Stockholm, Stockholm County, Sweden; Department of Epidemiology, Second Medical Faculty, Charles University Prague, Prague, Czech Republic

**Keywords:** Birth characteristics, Dementia risk, Demographic risk factors, Early life

## Abstract

**Objectives:**

Very early-life conditions are recognized as critical for healthy brain development. This study assesses early-life risk factors for developing dementia. In the absence of historical medical birth records, we leverage an alternative full population approach using demographic characteristics obtained from administrative data to derive proxy indicators for birth complications and unfavorable birth outcomes. We use proxy variables to investigate the impact of early-life risk factors on dementia risk.

**Methods:**

We use administrative individual-level data for full cohorts born 1932–1950 in Sweden with multigenerational linkages. Records on hospitalization and mortality are used to identify dementia cases. We derive 3 birth risk factors based on demographic characteristics: advanced maternal age, narrow sibling spacing, and twin births, and apply survival analysis to evaluate long-term effects on dementia risk. We control for confounding using multiple indicators for socio-economic status (SES), including parental surnames, and by implementing a sibling design. As comparison exposure, we add low education from the 1970 Census.

**Results:**

The presence of at least 1 birth risk factor increases dementia risk (HR = 1.059; 95% CI: 1.034, 1.085). The occurrence of twin births poses a particularly heightened risk (HR = 1.166; 95% CI: 1.084, 1.255).

**Discussion:**

Improvements to the very early-life environment hold significant potential to mitigate dementia risk. A comparison to the influence of low education on dementia (the largest known modifiable risk factor) suggests that demographic birth characteristics are of relevant effect sizes. Our findings underscore the relevance of providing assistance for births experiencing complications and adverse health outcomes to reduce dementia cases.

In light of the limited progress regarding disease-modifying medical treatments for Alzheimer’s disease and related dementias (ADRD), the Lancet 2020 commission underscores the importance of prevention targeting modifiable risk factors (Livingston et al., 2020). Existing research on dementia prevention largely focuses on mid-life risk factors (Xu et al., 2015), with limited attention to other possibly critical periods in life, including the very early-life period which encompasses intrauterine development and delivery. Directing our attention to this early period and truly adopting a life-course perspective has the potential to unveil novel risk factors and provide guidance for future preventative and therapeutic efforts (Pini & Wennberg, 2022).

The objective of this paper is to assess early-life risk factors for developing dementia. Research on this topic is limited. First, there are studies examining the observational relationship between birth characteristics and later-life cognitive abilities. This research relies on small sample sizes and is often of limited generalizability, due to reliance on birth records from specific health care centers. Krishna et al. (2022) recently showed that weight and length at birth are associated with cognition in later life among circa 721 adults in India. Some other studies focusing on cognitive abilities as an outcome (Erickson et al., 2010; Heinonen et al., 2015; Martyn et al., 1996; Shenkin et al., 2009) similarly rely on small sample sizes and provide mixed results. Second, an even more limited literature focuses on the importance of birth characteristics for ADRD. Mosing et al. (2018) investigate a large sample of twin pairs (*N* = 35,191) and identify an inverse association between birth weight and dementia risk, as well as a positive association between smaller head circumference and dementia risk. However, caution should be exercised in generalizing these findings to the broader population due to the specific characteristics of the study sample relying on twins only. In sum, evidence linking very early-life characteristics to dementia risk remains sparse.

There are several reasons as to why birth characteristics may exert an influence on the development of dementia in later life. Birth characteristics, such as birth weight, length, or head circumference, are all indicators of intrauterine fetal growth and development. Thus, one reason for a relationship with dementia may be that slow fetal growth and intrauterine development lead to differences in brain structure and function that persist into old age. For example, men who experienced undernutrition in early gestation following exposure to the Dutch famine during World War II had smaller total brain volumes (de Rooij et al., 2016) and worse brain perfusion (de Rooij et al., 2019). Similarly, Wheater et al. (2021) show that birth weight is associated with brain tissue volumes in old age, but not with MRI markers of neurodegeneration.

Recently, it was also reported that birth weight shows a remarkably consistent spatial pattern to cortical volumes across children, middle-aged, and older adults, but no consistent relations to brain change (Walhovd et al., 2023). Part of the effect is likely environmental, as birth weight differences between monozygotic twins relate to brain structure (Casey et al., 2017; Nadig et al., 2021; Walhovd et al., 2023). Thus, early-life conditions may lead to individual differences in brain characteristics that remain stable into older age. When aging-related decline sets in, these individual differences in level may affect the age at which critical thresholds (e.g., for functional independence and dementia diagnosis) are reached, even if cognitive and brain changes in older age are not affected per se (i.e., brain reserve; Satz, 1993). Alternatively, optimal early brain development may positively influence the acquisition of cognitive and psychosocial skills during development—skills that may make cognitive performance in older age less sensitive to aging-related brain changes (i.e., cognitive reserve; Stern et al., 2023).

Birth characteristics like weight and length at birth are also associated with an increased risk of preterm birth and birth complications. A systematic review and meta-analysis of studies on humans demonstrates that preterm birth is associated with volumetric, morphologic, and microstructural alterations in the brain in adulthood (Kelly et al., 2023). The studied birth characteristics are also associated with birth complications, such as perinatal asphyxia or hypoxic-ischemic encephalopathy. These complications commonly affect cognitive function and the brain of the child and may also influence the development of ADRD (Nalivaeva et al., 2018; Tarkowska, 2021). For example, Shen et al. (2020) use a mouse model and suggest that antenatal hypoxia, which decreases brain mass and body weight in the postnatal period, accelerates the onset of Alzheimer’s disease pathology, with cognitive decline and higher synaptic loss despite the recovery in brain and body mass in later life. Henceforth, the presence of birth complications may serve as a supplementary explanatory factor for the observed association between birth characteristics and ADRD.

A key hindrance faced by prior research efforts investigating the link between early-life risk factors and dementia was the lack of comprehensive historical data on birth outcomes for larger populations. The present study presents new insights into the intricate interplay between early-life factors and dementia risk, using an alternative approach that extends to a full population analysis to answer the research question of whether early-life risk factors have an impact on dementia. We use administrative data and demographic variables to assess very early life. Specifically, we examine three demographic risk factors—maternal age, birth spacing, and twin births. These birth-related characteristics were either previously examined in the literature or are closely linked to characteristics studied in smaller samples using data from health care centers. We hypothesize that the three risk factors, which are associated with lower birth weight, length, and birth complications, are associated with higher dementia risk. The present study advances prior research by using a sizeable sample (*n* = 1,568,049) that is representative of the elderly population in Sweden, resulting in more generalizable and precise estimates. Furthermore, we have sufficient power to investigate potential nonlinear effects of maternal age with dementia and can control for a comprehensive set of parental SES characteristics, which may confound the relationships of interest. We address potential confounding caused by biological or sociodemographic factors (or both), using a large set of background variables from multigenerational registers, and employing a sibling design.

## Methods and Materials

### Data

We use full population individual-level administrative data to examine the effect of demographic birth characteristics on dementia risk. The data is drawn from the Swedish Interdisciplinary Panel (SIP) covering the total population born 1930–1985 and their parents, linking several population registers including the Educational Register (1985–2016), the 1950, 1960, and 1970 Censuses, the Cause of Death Register (CDR; 1961–2016), and the Inpatient Hospitalization Register (1964–2016). Individuals are linked to their parents through the multigenerational Register, available for cohorts born in 1932 or later.

### Main Outcome

Following register-based research on dementia risk (Mosing et al., 2018; Seblova et al., 2021), dementia diagnoses are identified in the National Inpatient Register and the CDR using International Classification of Diseases codes (version 10 codes: F00, F01, F02, F03, and G30). Individual are followed from 1997 to the end of 2016 and an individual is classified as having dementia if the main or any secondary diagnosis for hospitalization or cause of death includes dementia. As a proxy for the onset timing of dementia, we use the earliest date of hospitalization or date of death. We focus on overall dementia and do not examine dementia by type as an outcome. Appendix Table A1 shows that most diagnoses (54.92%) in our sample are unspecific dementia cases (ICD-10 F03) and low subtype specificity prevents meaningful conclusions without assumptions about their distribution (Rizzuto et al., 2018).

Leveraging health administrative data to identify dementia cases enables the analysis of large sample sizes, required when applying proxy indicators. However, a trade-off exists in terms of moderate sensitivity, with the cohorts studied herein detecting only 70% of all dementia cases (Rizzuto et al., 2018; Seblova et al., 2021). The true timing of dementia onset likely occurs before when it is measured by hospitalization or death, whereby our outcome measure likely captures more severe cases of dementia.

### Exposure

Actual birth characteristics data for the full population is only available from 1973 in Sweden. We derive risk factors for birth complications and adverse birth outcomes using demographic family links. Our choice of characteristics is driven by empirical evidence from pediatrics and epidemiological research. Using linked multigenerational data, we identify the following risk markers for pregnancy and birth complications:

1. Advanced maternal age proxied by a binary indicator of high maternal age (maternal age at birth *≥*35) or in 3-year age intervals,
2. Short inter-pregnancy interval for an index sibling, capturing a narrow (*≤*18 months) spacing to the next oldest sibling, and
3. Twin/multiple births, measured as a binary indicator.

All of the above risk factors are associated with birth complications and adverse birth outcomes. Meta-analytic estimates based on 46 studies indicate that advanced maternal age (*≥*35) is associated with a 45% increase in preterm births (Lean et al., 2017). Additionally, advanced maternal age is associated with infants born being small for gestational age (25 studies, 16% increased risk), of low birth weight (35 studies, 37% increased risk), having fetal growth restriction (12 studies, 23% increased risk), and increased risk for being admitted to neonatal intensive care (NICU) admission (20 studies, 49% increased risk) suggesting a rise in the incidence of birth complications (Lean et al., 2017). A meta-analysis demonstrates that a short inter-pregnancy interval is linked to a 50% increased risk of preterm birth, a 33% increased risk of low birth weight, and poorer fetal growth with a 14% increased risk of newborn being small for gestational age (Wang et al., 2022). Short inter-pregnancy interval also is associated with a 25% increased risk of being admitted to NICU (Wang et al., 2022). Twin births are associated with a high risk of complications during pregnancy and childbirth, with more NICU admissions, and more than five times increased risk of stillbirth compared to singleton pregnancies (Cheong-See et al., 2016). Fetal growth restriction is also more common in the case of twin pregnancies with incidence rates varying between 16% and 48% across studies (Townsend & Khalil, 2018), and according to a Finnish study about half of all twin births were preterm deliveries (Rissanen et al., 2019). In sum, twin pregnancies are at a heightened risk for unfavorable pregnancy outcomes and birth complications.

### Covariates

In the empirical analysis, we include several indicators for parental SES. First, we construct a high-SES indicator, proxied by the household head being an entrepreneur or manager, using the socioeconomic group variable (SEI, socioekonomisk indelning) in the 1960 Census. Based on SEI, we further construct an indicator for the household head being a farmer. Parental education is also derived from the 1960 Census and refers to whether the household head has at least upper secondary education. Finally, we include an indicator of patronymic surnames, which captures low socioeconomic status (Clark et al., 2015). Research indicates that surnames have strong social origins (Hanks et al., 2016) and economists and historians use them when parental SES data is scarce. As an additional covariate, we include birth order for those that have a sibling in the empirical analysis to control for possible impacts on dementia risk from being first versus later born.

### Sample Selection

The baseline population consists of all individuals born in Sweden 1932–1950, excluding individuals emigrating or dying before 1961 (*N* = 1,924,191). We exclude individuals without proper linkage to a biological mother (*N* = 190,329) as this information is necessary to construct the demographic birth risk factors of interest. We also exclude individuals with unreasonably short birth spacing of less than 10 months (*N* = 286) and individuals who died or emigrated before the age of 65 (*N* = 165,527). Our final sample consists of *N* = 1,568,049 observations. Appendix Figure A2 shows the construction of our sample from the baseline population of all individuals born in Sweden 1932–1950 as a flowchart. The individuals in our study enter the sample at age 65 and are followed until administrative censoring in 2016, which is the last year available in the SIP data. Appendix Figure A3 shows the exact sample structure. The median follow-up time is 7.5 years. Our sample covers pre- and post-World War II cohorts. Neutral Sweden saw a birth rate rise in the mid-1940s but had a fertility recovery already from the mid-1930s. Education-related fertility gaps lessened during the baby boom, as educated women had younger first births (Stanfors, 2003). Fertility behavior also aligned across SES groups, establishing a two-child norm (Sandström & Marklund, 2017).

### Main Statistical Analysis

We estimate the associations between very early-life risk factors and the risk of dementia using a Cox proportional hazards model within a survival model framework:


$$\begin{array}{l} \lambda \left(t|X,\;Z\right)\;=\;\lambda_{0}\left(t\right)\;exp\left(\gamma_{1}Z_{i}\;+\;{\gamma_{2}}^{\prime }X_{i}\right), \end{array}$$ (1)

where *X*_*i*_ is a vector of control variables for potential confounding (high parental SES, parental education, patronymic surnames, farmers, birth order, sex, and birth year) and where *Z*_*i*_ represents a very early-life risk factor. λ_0_(*t*) represents the baseline hazard. As the occurrence of a birth risk factor does not necessarily imply birth complications nor adverse birth outcomes, we interpret γ_1_ analog to an intent-to-treat parameter as a lower bound for the association between birth complications or adverse birth outcomes on dementia risk. Testing proportional hazards assumption reveals violations for some birth cohort indicators and sex, but no violations for any of the exposures. We run proportional hazard models stratified by birth cohort and sex which deliver identical results without violations of the proportional hazards assumption.

### Sensitivity and Additional Analyses

To mitigate possible omitted variable bias, we estimate Cox proportional hazards models with sibling-fixed effects as an additional analysis. These models can control for unobserved confounding factors that are commonly shared among siblings, such as family background, genetics, and upbringing. In these models, the baseline hazard is stratified by the biological mother. As discussed by Kravdal (2019), this approach introduces linear dependence between birth year and maternal age in the within-family design. Functional form assumptions are a possible way to identify effects in otherwise collinear models (Blanchflower & Oswald, 2008; McKenzie, 2006). Our identification of effects stems from an implicit functional form assumption on the effect of maternal age on dementia by comparing mothers above and below aged 35 only.

To quantify the magnitude of our estimated associations and anchor effect sizes, we compare the point estimates for demographic birth characteristics to the association between low education and dementia risk (for both the full sample and the sibling sample), with low education being regarded as the single largest, modifiable risk factor in preventing dementia (Livingston et al., 2020). Educational attainment is derived from the 1970 Census and low education refers to having less than nine years of schooling (primary school only). This exercise allows us to qualify the clinical relevance of our estimates in the presence of a potential attenuation bias from moderate sensitivity in our outcome variable and outcome misclassification. We intentionally do not estimate the joint contribution of risk factors and education by adding lower education as an additional control variable. The main concern is the inclusion of *bad control* variables for our exposures by controlling for a mediator and introducing an *overcontrol bias* (Angrist & Pischke, 2009; Pearl, 2009).

We make complementary analyses and test the sensitivity of the baseline results, including adding parity as a risk factor, using an alternative definition of dementia, estimating the relationship of interest using logistics regressions, and testing the relationship between risk factors and infant and child mortality, respectively. We also provide evidence to show that our proxy variables strongly are associated with birth complications and adverse health outcomes at birth for younger cohorts.

## Results

**Figure 1.**
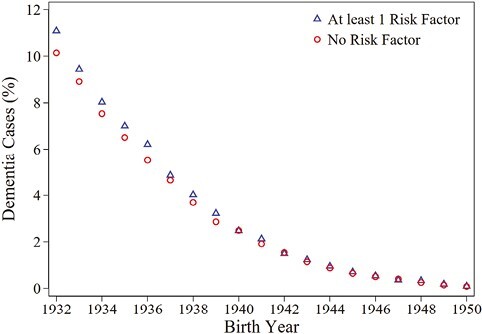
Risk Factors and Dementia Risk. The figure shows the share of individuals having at least one dementia diagnosis for the years 1997–2016 by very early-life risk factors. Results refer to cohorts born 1932–1950, having a mother in the multigenerational register. Risk factors are twin birth, maternal age ≥35, or sibling spacing ≤18 months. Source: SIP. Own calculations.

Descriptive statistics in Table 1 show that 2.35% of all individuals in our sample had at least one dementia diagnosis between 1997 and 2016 (36,797 out of 1,568,049). For the maximum follow-up of 20 years, 11% have at least one dementia diagnosis. Unadjusted, the risk of dementia is 0.1 percentage points higher in the group with at least one demographic birth risk factor (*p* < .01, relative risk 1.043). Figure 1 shows the declining risk of dementia by birth cohort, with a higher risk with the occurrence of at least one of the demographic birth risk factors. Table 1 also shows a correlation between lower socioeconomic status and maternal demographic birth risk factors. The occurrence of at least one risk factor is associated with a family background as farmers, and low SES based on parental occupation, lower parental education, and patronymic surnames, respectively.

**Table 1.** Descriptive Statistics

Variables	(1)	(2)	(3)	(4)	(5)
	Total	No risk factor	≥1 Risk factor	Δ = (3)-(2)	*p* Value Δ
Background					
Year of birth	1942.44	1942.43	1942.48	0.06	.00
Male (%)	49.71	49.76	49.55	−0.21	.02
Background farmer (%)	17.79	16.28	22.32	6.04	.00
Background high SES (%)	12.59	13.04	11.24	−1.80	.00
Background high Educ. (%)	7.37	7.65	6.53	−1.11	.00
Surname low SES (%)	47.68	47.10	49.41	2.31	.00
Risk factors					
≤18 months birth space (%)	6.00	0.00	23.82	23.82	.00
Twin birth (%)	1.84	0.00	7.31	7.31	.00
Age mother at birth ≥35 (%)	18.67	0.00	74.12	74.12	.00
Age mother at birth	28.59	26.43	35.04	8.61	.00
Only primary school (%)	49.44	48.35	52.69	4.34	.00
≤18 months birth space (%)	6.00	0.00	23.82	23.82	.00
Dementia outcomes					
Dementia diagnosis (1985–2016) (%)	2.35	2.32	2.42	0.10	.00
Age at first diagnosis	75.02	75.06	74.90	−0.16	.00
***N***	1,568,049	1,173,142	394,907	1,568,049	1,568,049

*Notes*: SES = socio-economic status. Column 1 shows mean values for the total study population. Columns 2 and 3 split the subpopulation by having none of the 3 early-life risk factors or having at least 1 risk factor. Risk factors are twin birth, maternal age ≥35, or sibling spacing ≤18 months. Column 4 gives the mean difference ∆between study individuals with and without early-life risk factors and Column 5 the *p* value for testing if the mean difference between both groups is different from zero (*H*_0_: ∆ = 0). Source: SIP. Own calculations.

Panel A in Table 2 shows our main regression results. Adjusting for sex and birth cohort, the occurrence of at least one risk factor is associated with a 5.9% increase in the hazard of being diagnosed with dementia (HR = 1.059; 95% CI: 1.034, 1.085). When adjusting for the socioeconomic status of the parents (parental education, SES based on occupation, patronymic surname, and farmer) and birth order, the association decreases to a 5.2% increase in the hazard (HR = 1.052; 95% CI: 1.027, 1.079). To control for additional confounding within a family not accounted for by our measures for socioeconomic status, we estimate a sibling design with stratified baseline hazard by biological mother. The point estimate in Column (3) remains consistent with the baseline estimates, which lends credence to the notion that SES does not substantially confound our findings. Given that the sibling design requires within-sibling variation in exposure, our additional estimates are less precise than the baseline results and we cannot reject the null hypothesis of no increased hazard at the 5% significance level (HR = 1.059; 95% CI: 0.998, 1.124).

**Table 2.** Main Regression Results

Exposure	(1)	(2)	(3)
	Base	Adjusted	Within-family
A: Combined risk factors			
≥1 early-life risk factor	1.059	1.052	1.059
	[1.034,1.085]	[1.027,1.079]	[0.998,1.124]
B: Separate risk factors			
Twin birth	1.166	1.171	1.225
	[1.084,1.255]	[1.088,1.261]	[1.019,1.473]
Age mother at birth ≥35	1.049	1.040	1.072
	[1.022,1.076]	[1.013,1.068]	[0.982,1.169]
≤18 months birth space	1.067	1.068	1.018
	[1.017,1.120]	[1.017,1.121]	[0.946,1.096]
C: Low education			
<9 Years of school	1.239	1.232	1.079
	[1.212,1.267]	[1.203,1.262]	[1.015,1.147]

*Notes*: CI = confidence interval; HR = hazard ratio. The table shows associations between selected risk factors and the risk of dementia diagnosis. Dementia follow-up was between 1997 and 2016. Results refer to cohorts born 1932–1950, having a mother in the multigenerational register. Risk factors are twin birth, maternal age ≥35, or sibling spacing <18 months. Method: Proportional Hazard Model. All regressions control for cohort and sex by including dummy variables. Adjusted regressions add a set of control variables for socioeconomic status (high parental education, high occupational status, patronymic surnames, and farmers) and birth order. Within-family estimates are stratified by biological mother. Effects represent HR with 95% CI. Effects from own low educational status is given as comparison (anchor) for effect size. Source: SIP. Own calculations.

**Figure 2.**
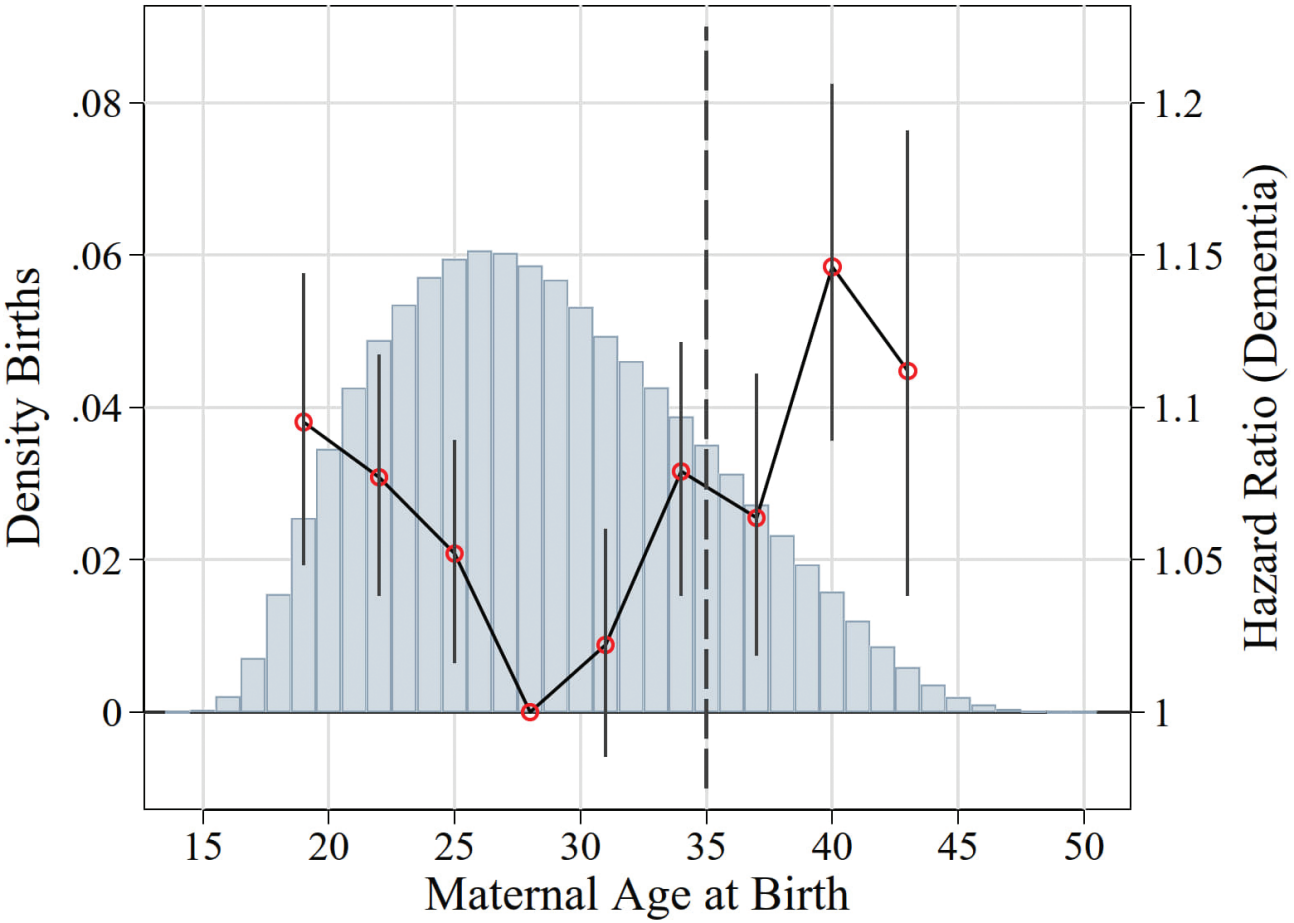
Nonlinearity maternal age. The figure shows associations between maternal age and the risk of dementia diagnosis along the maternal age distribution. Dementia follow-up was between 1997 and 2016. Results refer to cohorts born 1932–1950, having a mother in the multigenerational register. Maternal age was binned into 3-year indicators. Method: Proportional Hazard Model, controlling for cohort, birth order and sex, included as dummy variables. Effects represent HR with 95% CI. CI = confidence interval; HR = hazard ratio. Source: SIP. Own calculations.

Panel B in Table 2 presents the associations for each of the three demographic birth risk factors and dementia separately. Results in Column (1) of Panel B show heterogeneity between the risk factors, with twin birth giving the largest increase (16.6%) in the hazard of being diagnosed with dementia (HR = 1.166; 95% CI: 1.084, 1.255), followed by a 6.7% increase in the hazard from the short inter-pregnancy interval (HR = 1.067; 95% CI: 1.017, 1.120). Advanced maternal age only increases the hazard by 4.9% (HR = 1.049; 95% CI: 1.022, 1.076). Because the risk indicator for advanced maternal age is quite broad (age > 35), we also investigate potential heterogeneity by estimating the association between maternal age and the hazard of dementia using measures of maternal age at birth in 3-year bins. As shown in Figure 2, the risk of dementia appears to rise for both younger and older mothers in comparison to the reference category of mothers aged 24–27 at the time of childbirth. Column 2 shows that the separate association between dementia and all three risk factors is stable when controlling for SES. For the sibling design in Column 3, point estimates for twin births and advanced maternal age are slightly larger, while the estimated HR for the short inter-pregnancy interval is close to 1 and precision is lower.

**Figure 3.**
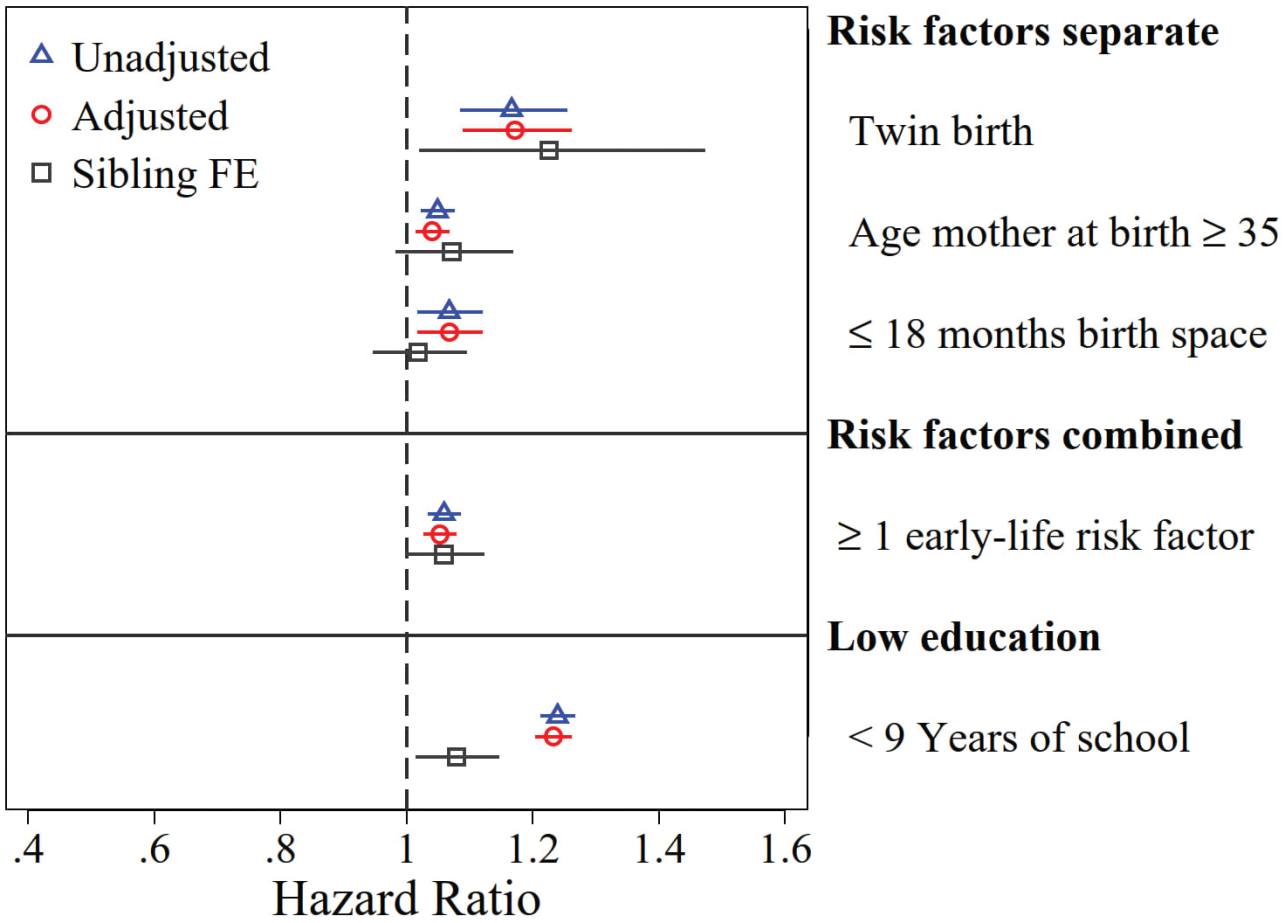
Regression results. The figure shows associations between selected risk factors and the risk of dementia diagnosis. Dementia follow-up was between 1985 and 2016. Results refer to cohorts born 1932–1950, having a mother in the multigenerational register. Risk factors are twin birth, maternal age ≥35, or sibling spacing ≤18 months. Method: Proportional Hazard Model. All regressions control for cohort, birth order and sex by including dummy variables. Adjusted regressions add a set of control variables for socioeconomic status (high parental education, high occupational status, patronymic surnames, and farmers). Effects represent HR with 95% CI. Effects from own low educational status is given as comparison (anchor) for effect size. CI = confidence interval; FE = fixed effects; HR = hazard ratio. Source: SIP. Own calculations.

Panel C in Table 2 shows our anchoring comparison focusing on low education, indicating a 23.9% (HR = 1.239; 95% CI: 1.212, 1.267) increased hazard of being diagnosed with dementia for individuals with only primary education (>9 years). As shown in Figure 3, the magnitude of the association between the presence of at least one demographic birth attribute and dementia corresponds at least to 1/4 of the magnitude of the association between low education and dementia. The association between low education and dementia is further sensitive to within-sibling comparisons. With stratification by biological mother, the hazard ratio decreases to 1.079 (95% CI: 1.015, 1.147).

When restricting our endpoint to individuals with dementia diagnosis only from inpatient registers, excluding mortality cases, the results barely change, see Appendix Table B1. This follows from the fact that only a minority of dementia cases are solely identified from the CDR. We next test robustness to modeling choice. Appendix Table B2 compares the survival model with binary choice models using every diagnosed with dementia as an outcome. Notably, timing does not affect the results and findings using the proportional hazard model and logistic regression, respectively, are essentially identical. This result can be backed up by prior research showing that Cox regressions and logistic regressions deliver similar results if the event probability is small and effect sizes are also small or moderate (Callas et al., 1998).

Finally, we also examine the impact of parity as a risk factor. Research suggests an impact of parity on dementia risk for parents (Bonsang & Skirbekk, 2022; Read & Grundy, 2017; Zhang et al., 2023), but medical research also suggests that null-parity has a higher risk of low birth weights and preterm birth, while evidence is mixed regarding the risk of being of high parity of five or more births (Koullali et al., 2020). Appendix Figure B2 shows evidence in line with this research, but notably, our data is not ideal to testing the impact of parity on dementia risk as we do not have information on complete parity for all mothers.

## Discussion

We examined the relationship between proxy measures of pregnancy complications and adverse birth health outcomes—advanced maternal age, short inter-pregnancy interval, and twin birth—on dementia risk in a population sample of Swedish individuals. Our main results indicate substantial associations between very early-life risk factors and the risk of dementia, with 5%–16% increased risk, mirroring substantial potential for reducing dementia burden. It is important to acknowledge that our risk proxies serve as indicators of potential adverse outcomes rather than direct targets for intervention. The focus of intervention should be on enhancing ante and postnatal care to mitigate the consequences associated with elevated levels of risk.

The interpretation of the magnitude of our estimates is challenging for two reasons. First, the presence of a demographic birth risk factor according to our definition does not automatically lead to adverse birth outcomes or complications. This also indicates that the potential role of adverse health outcomes at birth and complications is indeed larger than what is indicated by our estimates. The interpretation of our results thus analogs the interpretation of an intent-to-treat parameter. Second, we rely on dementia diagnosis from Inpatient and Cause-of-death administrative data which exhibit a certain lag with respect to the onset of dementia and where there is a potential for misclassification, further attenuating our estimates.

To gain a better understanding of the magnitude of our estimated effects, we apply two strategies. First, we compare our results to the role of low education, which is the largest single modifiable risk factor for later-life dementia (Livingston et al., 2020). The association of the studied demographic birth characteristics corresponds to at least a quarter of the magnitude of the relationship between low education and dementia. This relative effect size even becomes close to one when using the sibling design. For twin births, the magnitude of the association corresponds to 70% of the relationship between low education and dementia. One should also note that parts of the association between education, cognitive functioning, and late-life dementia may actually arise from early-life circumstances. Such circumstances can indirectly, through an effect on cognitive performance, affect educational attainment and risk of dementia in older age. These anchoring comparisons suggest that the role of the demographic birth characteristics is meaningful. They also suggest there is a large potential for intervention, not least by placing attention on improvements in antenatal and neonatal care. Again, we note that this relates to the consequences arising from birth risk factors rather than avoiding our proxy variables.

Second, we can compare our estimates to the study from Mosing et al. (2018), who use the Swedish twin registry, and is a rare exception in research on birth health outcomes and dementia diagnosis. Their estimates show that low birth weight is associated with an increase in dementia risk of 20%. In Appendix Figure B3, we show for more recent cohorts, estimates corresponding to an increase of about 23% (OR = 3.5) in low birth weight if at least one of our risk factors is present. Combining these results, a back-on-the-envelope calculation suggests an increase of 4.6% in dementia risk from one of the three risk factors, which in turn is very close to our estimates. Our results showing an elevated risk for twins to develop dementia also imply that the chosen sample of twins in Mosing et al. (2018) exhibits a greater baseline risk of developing dementia than the overall population.

Previous literature shows that the demographic birth risk factors used in this study correlate with socioeconomic background (Hutcheon et al., 2019; Molina-García et al., 2019). Controlling for a rich set of household SES covariates and estimating within-sibling comparisons did not substantially alter our results. An exception are the effects from short birth interval which are attenuated in the sibling design. Adding placebo tests for younger and older siblings of the index birth, we find no effects for unaffected siblings, but also attenuated effects on the restricted subsample of families with at least one short birth interval, indicating at least partial confounding (Appendix Figure B7). Overall, we conclude that it is unlikely that our results are primarily driven by confounding from SES. In contrast, estimates from the sibling design show a substantial attenuation for the relationship between low education and dementia. Controlling for unmeasured shared social and environmental family characteristics among siblings consequently captures a substantial part of the effect of low education. Therefore, in our study, the relationship between low education and dementia is more driven by SES confounding than the proxies for birth risks. These results further underscore the large potential of early interventions.

We interpret our results for demographic birth characteristics as effects arising from pregnancy complications and adverse birth health. At the same time, we do now know how frequently these characteristics lead to birth complications or adverse birth events for our study population. In fact, a major advantage of our design is that we estimate the effects on dementia risk in the absence of detailed historical data on birth outcomes. To determine the frequency at which pregnancy complications and adverse birth health outcomes are associated with our proxy variables to help interpretation in case of an intent-to-treat parameter, we investigate the relationship using current birth records pertaining to cohorts born between 1973 and 1985. Appendix Figure B3 shows that our proxy variables are associated strongly with birth complications and adverse health outcomes at birth. Appendix Figure B4 shows the same for the nonlinear association in maternal age. A caveat with this analysis is that medical practices, guidelines, and therapeutic options have changed over time which in turn affects inference regarding the relationship between risk factors and actual birth complications in our older cohorts. Given that practices have improved, and medical assistance better tailored to handle and prevent birth complications, we however expected an even stronger association between birth characteristics and birth outcomes in the past. Furthermore, we need to assume that the main effect operates through birth outcomes and not through other effects as the family composition changes due to the presence of twins or older parents. This is ultimately an untestable assumption and inherent in our proxy approach. Furthermore, it is possible that part of the effect is mediated through mid-life factors such as education or income. We intentionally do not differentiate between direct and indirect effects to avoid an overcontrol bias. Appendix Figure A1 shows in detail the different potential pathways and how our risk factors could relate to dementia as an outcome.

As an alternative approach to examining the validity of our exposures, we examine the association between one of our identified risk factors with childhood mortality. If the identified exposures are good proxies for insults to fetal development, this exercise should reveal an association with infant and, possibly, child mortality. For twins, we can combine the complete 1950 Population Census and the Swedish Death Registry, including all deaths in Sweden 1901–2013, which allows us to identify all twins. As the data cover the whole universe of the population (minus emigrants), we can estimate the effect of being a twin on infant and child mortality, respectively, for the cohorts born 1932–1950. Appendix Figure B5 shows higher infant mortality in twin births while early childhood mortality is less affected.

A major strength of our study is that we can assess the role of very early-life factors in dementia in late life in the absence of detailed historical birth records by exploring full administrative population data and using proxy measures of pregnancy complications and adverse birth health outcomes. These proxies (twin birth, maternal age, and spacing) are further measured without any substantial error in the registers. Our findings suggest that administrative demographic data, available in many countries, can be used in future research to further provide insights into the complex interplay between very early-life factors and dementia. Another key strength of our study is that we have access to a broad set of control variables and also address confounding by a sibling-fixed approach.

Our study naturally has several weaknesses. First, our analysis cannot separate between the suggested underlying reasons for why the examined risk factors relate to dementia. For example, while twin birth is predictive of birth complications, twins also have more restricted fetal growth, whereby our estimates on dementia risk may reflect both these mechanisms. The exact causal chain between very early-life factors and dementia remains unknown. Our study can only draw conclusions regarding the association between birth risk factors and dementia in old age. Second, while leveraging administrative data enables an analysis of large sample sizes, our dementia measure based on the cause of hospitalization and death has moderate sensitivity and likely primarily captures more severe cases of dementia.

Finally, we need to discuss the external validity of our study. Clearly, given medical and scientific progress, the risk factors identified are likely less problematic today than during 1930–1950. For instance, in modern maternal-fetal health care, older pregnant women generally undergo systematic monitoring, while specialized programs address twin pregnancies, involving structured fetal growth assessments and amniotic fluid measurement (Socialstyrelsen, 2020). The full dementia impact of these risk factors on today’s cohorts remains uncertain, but we believe our findings have contemporary relevance as poor health affects around 200 million children under five in developing nations, hindering cognitive potential, and perpetuating poverty across generations (Bhalotra et al., 2022; Grantham-McGregor et al., 2007). Our research suggests enhancing early-life environments could reduce dementia risk, highlighting the need to aid births with complications for reducing dementia cases, especially in countries at a similar development stage to Sweden’s past.

External validity is also relevant for our sibling design effects, especially regarding the estimation of twin effects. Families in sibling design need at least three children to identify a twin effect, which may threaten their representativity. We find that the families are larger and that non-twin siblings are more often older. This suggests some expected adjustment of birth parity when parents receive twins. The within-family twin sample further has a 0.36% points higher dementia risk and lower SES (baseline 2.33%, statistically significant difference, but note that this higher risk is partially driven by the twin effect itself). If greater family size and lower SES are associated with an increased risk of dementia, then the within-family estimates can be expected to be larger due to a higher baseline risk. This is also what we find. Time may also constitute a threat to the external validity of our twin results. Earlier research showed that twins are vulnerable early in life for cognitive development for cohorts born before 1950, an effect that might be less relevant today (Christensen et al., 2006). The magnitude from within-family estimates for twins should therefore take potential different baseline risks into account.

In conclusion, our study suggests that improvements to the very early-life environment hold significant potential to substantially mitigate the risk of developing dementia. Our findings underscore that providing assistance for births experiencing complications and adverse health outcomes can be of relevance to reducing the number of dementia cases.

## Supplementary Material

gbad142_suppl_Supplementary_Appendix

## Data Availability

The administrative individual-level data is available for any researcher, but at a cost and given security rules for Swedish administrative data, they are only available in a secure remote access system and subject to an application process requiring ethical approval. Researchers using the data will be required to work from a country within the European Union. In view of the rules imposed by the Swedish Government, we will not be able to post the individual-level data if the paper is accepted for publication. Stata code, used to prepare the data and to run the analysis, will be published to allow future replication.

## Author Notes

1. For a majority of years, we have information regarding National Inpatient Register (NPR) diagnosis and cause of death on the three-digit level. For the years 2012–2016 in NPR, we also have more fine-grained data with four digits. It is preferable to identify dementia using version 10 codes F00.0, F00.1, F00.2, F00.9, F01.0, F01.1, F01.2, F01.3, F01.8, F01.9, F02.0, F02.3, F03.9, G30.1, G30.8, G30.9, G31.1, and G31.8A. We compare the two approaches for the period 2012–2016. Reassuringly, the three digits approach only misses 1% of all dementia cases. The minimal measurement error is unrelated to our exposure variables.

2. Appendix Table B6 shows a replication exercise comparing full population estimates for effects from education with the findings of based on SNAC-K data using clinical dementia assessment. The results suggest an attenuation bias of expected size given the lower sensitivity of our outcome measure. This bias should however be proportional for different regressors and cancels out when relative effects of difference regressors are considered as ratio.
