# Supplementary material for: Very Early-Life Risk Factors for Developing Dementia: Evidence From Full Population Registers
Source: J Gerontol B Psychol Sci Soc Sci. 2023 Sep 26;78(12):2131–40. doi: 10.1093/geronb/gbad142 (PMC10699746; doi:10.1093/geronb/gbad142)
Supplement: gbad142_suppl_Supplementary_Appendix [file gbad142_suppl_supplementary_appendix.pdf]

Very Early Life Risk Factors for Developing Dementia - Evidence from full population registers

Online Appendix

Appendix A Pathway Diagram and Descriptive Data

Figure A.1 presents a causal pathway diagram illustrating our conceptual framework regarding the relationship between our identified risk factors and dementia risk. Solid lines represent our suggested pathways, either direct or indirectly transmitted via intermediate outcomes. Notably, our identified risk factors might operate directly on intermediate outcomes and/or dementia risk. This still makes the identified risk factors a valid risk factor for dementia, but the overall explanation to why we find an effect may be different.

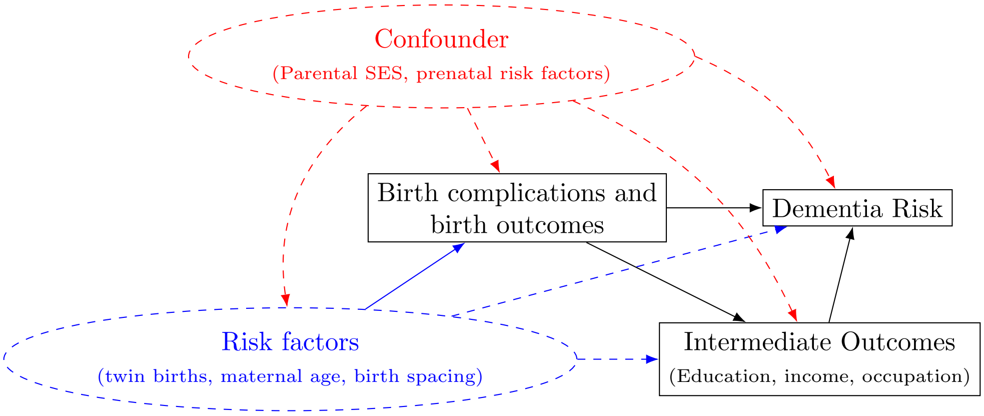

FIGURE A.1 *(Causal Pathway)*

Figure A.2 illustrates how we derive our study sample from the baseline population of all individuals born in Sweden 1932-1950. The individuals in our study enter the sample at age 65 and are followed until administrative censoring in 2016, which is the last year available in the SIP data. Figure A.3 illustrates the exact sample structure. The median follow up time is 7.5 years.

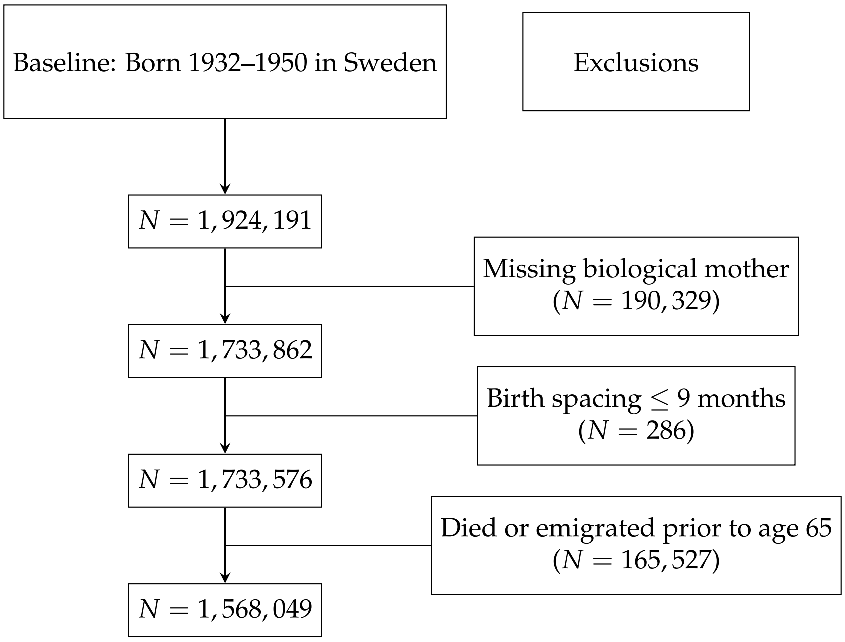

FIGURE A.2 *(Flow-chart sample restriction) Source:* SIP. Own calculations.

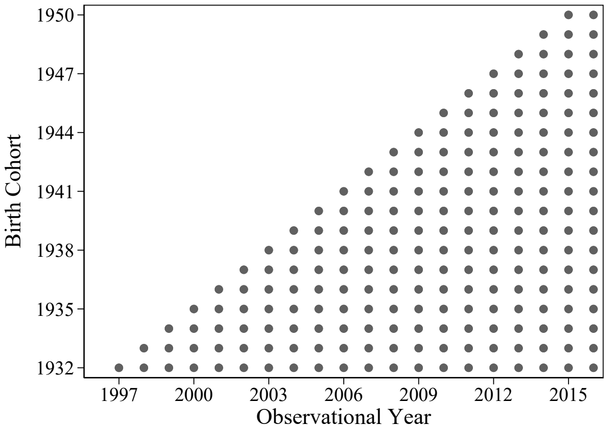

FIGURE A.3 *(Sample Structure)*: The figure shows the available year cohort combination in our data and follow up time for each cohort.

Table A.1 shows the distribution of dementia diagnoses in our data. Most diagnosis are unspecific dementia cases (ICD-10 F03). This likely follows from the fact that most dementia cases are secondary diagnosis of elderly individuals with a different main cause for hospitalisation or death. Their dementia state is thus correctly identified, but the underlying type of dementia must not be.

Table A.1: Dementia Frequency

Dementia Type	(1)	(2)	(3)	(4)	(5)	(6)
	Total		Cause of Death		Inpatient Registry	
	Freq.	%	Freq.	%	Freq.	%
F00	75294	12.45	10966	5.42	64328	15.98
F01	98698	16.32	28820	14.25	69878	17.36
F02	12163	2.01	1386	.69	10777	2.68
F03	332070	54.92	129102	63.85	202968	50.43
G30	86440	14.3	31914	15.78	54526	13.55

Notes: The table shows type of dementia diagnosis by ICD-10 Code in our data for years 1997–2016. The dementia codes refer to the following types: F00 Dementia in Alzheimer disease, F01 Vascular dementia, F02 Dementia in other diseases classified elsewhere, F03 Unspecified dementia, G30 Alzheimer disease*Source:* SIP. Own calculations.

While we know from previous research that we have high specificity in overall dementia diagnosis and basically zero false positives, as shown from cohort studies (Rizzuto et al., 2018), the specificity is much lower for the various sub-types being correctly identified. With 54.92% unspecific dementia cases we cannot draw conclusions from results on specific sub-types of dementia without further assumption on the distribution within the unclassified cases. Also, non-parametric bounds for the type of dementia are largely uninformative (Manski, 1990) and power will be low if analysing dementia sub-types. Based on the above, we only focus on overall dementia as an outcome as potentially interesting heterogeneity results by dementia type are difficult to interpret and requires more suitable data.

A strength of our analysis is that we can apply sibling design to examine the impact of identified risk factors on dementia risk. We believe this approach also is meaningful when examining twin births as exposure. First, by restricting the sample to families with twins and at least one additional sibling, we can show that we sufficient variation in twin exposure in families with multiple births and can compare the twins to other siblings. Table A.2 shows that we have 550 non-twin siblings with dementia and 455 twins with dementia in this restricted sample including a total of 37,216 individuals belonging to this subset of families. Power analysis reveals that in the within family sample we has a power close to 70% to detect effects from twin birth on dementia.[1] This power assessment is conservative as other observations contribute to an increased precision of our within estimates in the full sample, whereby we deem within family estimates to be enough powered.

[1]We use the baseline association of 0.166 as baseline effect, and the exact power is 68,23%.

Table A.2: Variation within family twins

Dementia diagnosis	Other Siblings	Twins	Total
0	17859	18352	36211
1	550	455	1005
Total	18409	18807	37216

Notes: The table the variation in within family exposure and twins. *Source:* SIP. Own calculations.

It should be noted that it is necessary to have at least two births and minimum of three children per mother in order to identify an effect when applying the siblings approach using twins as exposure. This shifts the sample contributing to identification of within family twin effects to the larger families as shown in Figure A.4. Table A.3 shows that these families have slightly lower SES and are more often farmers, but not fundamentally different in magnitude. The within family twin sample further has 0.36 percentage points higher dementia risk (baseline 2.33%, statistical significant difference). Notably this higher risk is partially driven by the twin effect itself. If greater family size / parity and lower SES associates with increased risk in dementia, then the within family estimates can be expected to be larger due to a higher baseline risk. Overall, apart from the increase in baseline dementia risk, the point estimates and background characteristics are similar in both samples.

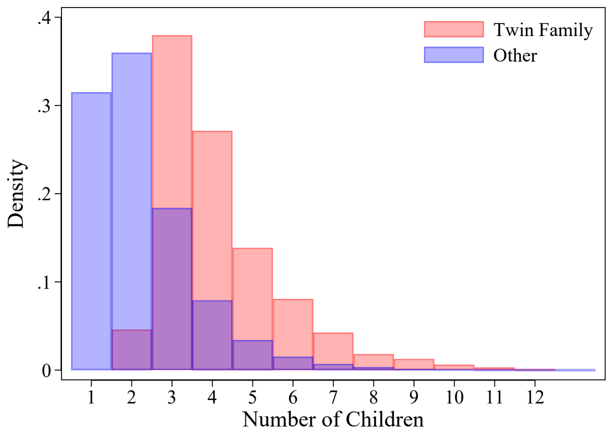

FIGURE A.4 *(Family Size)*: The figure shows the family divided by the presence of twins. Twin families have twins and further at least one further siblings. These families contribute to the identification of within family twin effects on dementia risk.

Table A.3: Descriptive Statistics: Within Twin Sample

	(1) Total	(2) Other	(3) Within Twin Sample	(4) $\Delta = (3)$ -(2)	(5) $p-$value Δ
BACKGROUND					
Year of Birth	1942.44	1942.45	1942.09	-0.36	0.00
Male (%)	49.71	49.72	49.31	-0.41	0.12
Backgrond Farmer (%)	17.79	17.60	25.67	8.07	0.00
Backgrond High SES (%)	12.59	12.61	11.64	-0.97	0.00
Backgrond High Educ. (%)	7.37	7.39	6.56	-0.83	0.00
Surname Low SES (%)	47.68	47.62	50.36	2.74	0.00
RISK FACTORS					
<=18 months birth space (%)	6.00	5.96	7.62	1.66	0.00
Twin Birth (%)	1.84	0.66	50.53	49.88	0.00
Age mother at birth > 35 (%)	18.67	18.59	21.79	3.20	0.00
Age mother at birth	28.59	28.56	29.66	1.09	0.00
Only Primary School (%)	49.44	49.22	58.57	9.35	0.00
DEMENTIA OUTCOMES					
Dementia Diagnosis (1985–2016) (%)	2.35	2.34	2.70	0.36	0.00
Age First Diagnosis	75.02	75.02	74.95	-0.06	0.67
N	1,568,049	1,530,833	37,216	1,568,049	1,568,049

Notes: Column 1 shows mean values for the total study population. Column 2 and 3 split the subpopulation by within twin sample. Within twin sample needs at least one other sibling which is not a multiple birth. Risk factors are twin birth, maternal age ≥ 35, or sibling spacing ≤ 18 months. Column 4 gives the mean difference Δ between study individuals with and without early life risk factors and column 5 the p-value for testing if the mean difference between both groups is different from zero ($H_0: \ \Delta = 0$).

Appendix B Additional Results

In the below we present additional empirical results, complementing and testing the robustness of our main analysis.

One potential concern using a register based approach using inpatient and death cause data to identify dementia is that it is not clear that the timing of the hospitalisation and cause-of-death codes correspond to actual onset of dementia. For mortality this may generate an under-count of dementia cases if decedents with dementia are classified with a non-dementia code. We therefore perform a robustness test to assess whether the exclusion of cases identified from cause of mortality alter the baseline results. When restricting our endpoint to individuals with dementia diagnosis only from inpatient registers, the results barely change, see Table B.1. This result follows from the fact that only a minority of our dementia cases is solely identified from the cause of death register.

Table B.1: Sensitivity Drop Cause of Death

	(1)<br>Base	(2)<br>w/o Cause of Death
Twin Birth	1.166	1.190
	[1.084,1.255]	[1.101,1.287]
Age mother at birth ≥ 35	1.049	1.040
	[1.022,1.076]	[1.012,1.070]
≤ 18 months birth space	1.067	1.066
	[1.017,1.120]	[1.012,1.123]

Notes: The table shows associations between selected risk factors and the risk of dementia diagnosis. Dementia follow up was between years 1985–2016. Results refer to cohorts born 1932 –1950, having a mother in the multi-generational register. Risk factors are twin birth, maternal age ≥ 35, or sibling spacing < 18 months. Method: Proportional Hazard Model. All regressions control for cohort and sex by including dummy variables. Effects represent HR with 95% CI. Column 2 restricts estimations to diagnosis only from inpatient data and treats individuals with only dementia diagnosis in the cause of death as censored observations. *Source:* SIP. Own calculations.

Next we test the robustness of our results with respect to modelling choice. We here compare the survival model with binary choice models using *ever diagnosed with dementia* as an outcome. Results in Table B.2 suggest as that the timing indeed does not affect the results in a meaningful shape or form. Results from the proportional hazard model and logistic regression are essentially identical (although HR and OR are not an exact match). This result is backed up by prior research suggesting that Cox regressions and logistic regressions deliver similar results if the event probability is small and effect sizes are also small or moderate (Callas et al., 1998).

Table B.2: Sensitivity Estimation Method

	(1) Proportional Hazard HR	(2) Logistic Regression OR	(3) Logistic Regression Marginal Effect	(4) LPM Marginal Effect
Twin Birth	1.166	1.171	0.004	0.004
	[1.084,1.255]	[1.086,1.263]	[0.002,0.005]	[0.002,0.006]
Age mother at birth $\geq$ 35	1.049	1.051	0.001	0.001
	[1.022,1.076]	[1.024,1.080]	[0.001,0.002]	[0.001,0.002]
$\leq$ 18 months birth space	1.067	1.063	0.001	0.002
	[1.017,1.120]	[1.012,1.117]	[0.000,0.002]	[0.001,0.003]

Notes: The table shows associations between selected risk factors and the risk of dementia diagnosis. Dementia follow up was between years 1985–2016. Results refer to cohorts born 1932 –1950, having a mother in the multi-generational register. Risk factors are twin birth, maternal age $\geq$ 35, or sibling spacing $<$ 18 months. Method: Proportional Hazard Model. All regressions control for cohort and sex by including dummy variables. Effects represent HR with 95% CI. Column 2 restricts estimations to diagnosis only from inpatient data and treats individuals with only dementia diagnosis in the cause of death as censored observations. *Source:* SIP. Own calculations.

A strength of our analysis is that we can apply sibling design to examine the impact of identified risk factors on dementia risk. At the same time this analysis demands careful interpretation, especially when it comes to twin birth exposure. Appendix A discusses requirements for identification and presents some descriptive statistics for the twin sample. Here we further compare the families of the siblings in the within twin sample. Table B.3 shows that the families are similar in their characteristics, but we have twice the number of older siblings then younger. This indeed speaks for some selection in terms of family size.

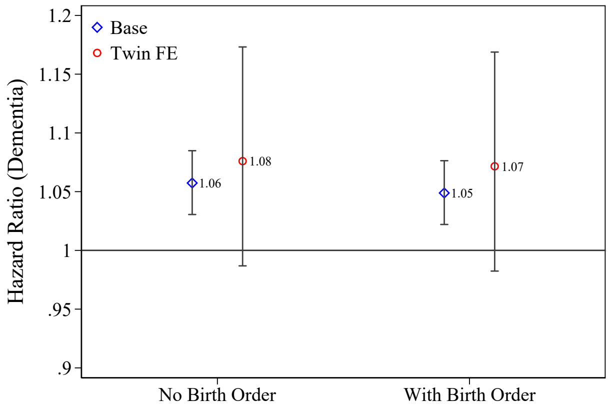

FIGURE B.1 *(Sensitivity to Birth Order Effects)*: The figure shows effects for maternal age (age at birth > 35) for specifications excluding and including birth order as additional control. We estimated base specification as well as the within family estimation.

Table B.3: Descriptive Statistics: Within Twin Sample Siblings

	Siblings	
	Younger	Older
Background		
Year of Birth	1944.54	1939.47
Male (%)	49.24	49.60
Backgrond Farmer (%)	28.63	26.49
Backgrond High SES (%)	10.08	11.41
Backgrond High Educ. (%)	5.31	6.12
Surname Low SES (%)	51.27	50.49
N	6,064	12,396

Notes: Table shows family background characteristics for siblings of twins.

Next we assess an alternative risk factor. There is a growing literature on the impact of parity on dementia risk for parents (Bonsang & Skirbekk, 2022; Read & Grundy, 2017; Zhang et al., 2023), but medical research also suggest that null-parity have a higher risk of low birth weights and pre-term birth, while evidence is mixed regarding the risk of being of high parity (+5) (see e.g. Koullali et al. (2020)). Figure B.2 presents results focusing on parity as a risk factor showing evidence in line with this research. At the same time, it is important to recall that our data set is not ideal to test the impact of parity on dementia risk as we do not have information on complete parity for all mothers, whereby we do not include these results in the main analysis.

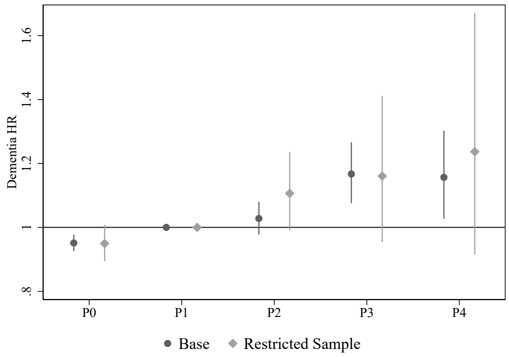

(a) Dementia (1932–1950)

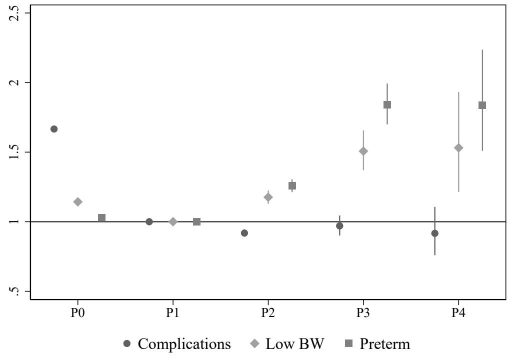

(b) Birth Outcomes (1973–1985)

Figure B.2 *(Risk factors and birth outcomes)*

Notes: Figures shows the association between birth risk factors and birth outcomes. As birth outcomes we measure birth complications as any maternal diagnosis indicating birth complications at birth (ICD 8 and 9, Codes 652-659), low birthweight (< 2500g), very low birthweight (< 1500g), preterm (< 37 weeks pregnancy) and very preterm birth (< 32 weeks of pregnancy). Method: Proportional hazard, logistic regression.
Source: SIP. Own calculations.

For the births in our study we miss data on actual birth complications and adverse birth outcomes. We argue that instead evidence can be presented using proxy variables which cap-

ture the risk for birth complications and adverse health outcomes at birth. To validate the choice of our measures, we estimate the relationship between the birth risk factors and health outcomes at birth on the first birth cohorts in the Swedish medical register. Figure B.3 shows the association between the three risk factors and complications at birth as well low birthweight and preterm births. Figure B.4 shows the non-linear association between health outcomes, complications at birth and maternal age.

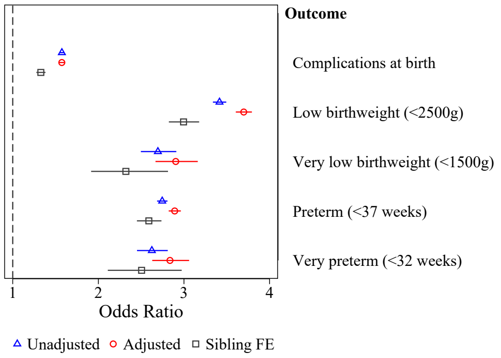

(a) ≥ 1 risk factor

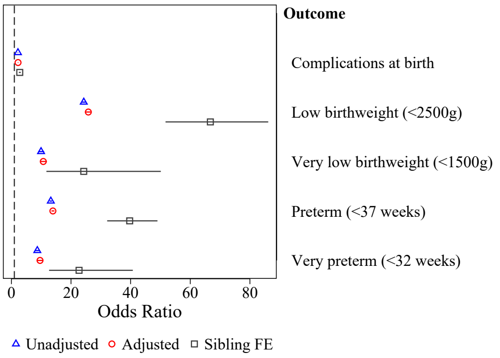

(b) Twins birth

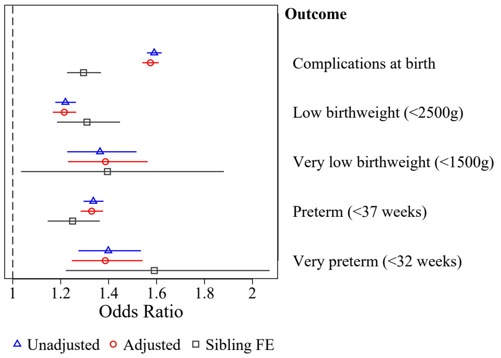

(c) Maternal age > 35

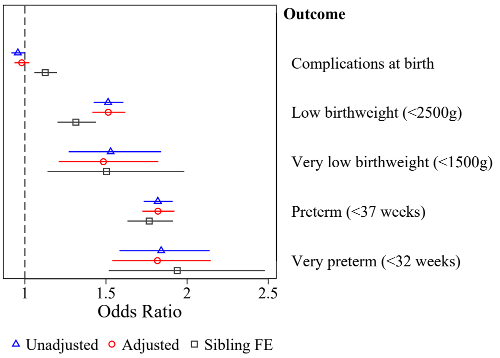

(d) ≤ 18 months birth space

FIGURE B.3 *(Risk factors and birth outcomes)*

Notes: Figures shows the association between birth risk factors and birth outcomes. As birth outcomes we measure birth complications as any maternal diagnosis indicating birth complications at birth (ICD 8 and 9, Codes 652-659), low birthweight ($<$ 2500g), very low birthweight ($<$ 1500g), preterm ($<$ 37 weeks pregnancy) and very preterm birth ($<$ 32 weeks of pregnancy). Method: Logistic regression. Adjusted regressions add a set of control variables for socio-economic status (parental education, income) and birth order. Maternal fixed effects are estimated by conditional logit regression. Estimates reflect odds ratios with 95% CI. Sample is restricted to children born 1973–1985, the first years available in the medical birth registry in Sweden.
Source: SIP. Own calculations.

An alternative approach to understand the validity of our identified risk exposures is to establish the associations between identified risk factors with mortality in childhood. If the identified exposures proxy for insults to fetal development, this exercises should reveal an associations with infant and/or child mortality. Depending on our the chosen exposure variable this exercise is differently challenging. For twins we can combine the complete 1950 Population Census and the Swedish Death Registry, including all deaths in Sweden 1901-2013. With

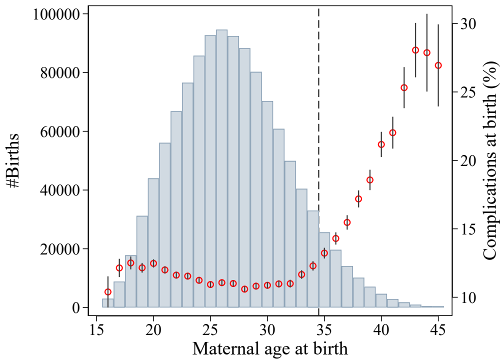

(a) Complications at birth

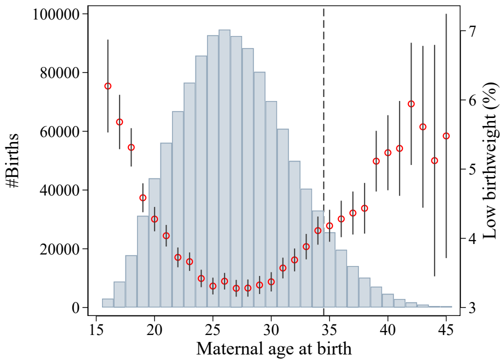

(b) Low birthweight

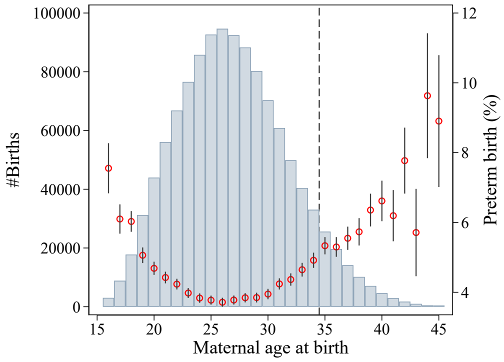

(c) Preterm birth

FIGURE B.4 *(Non-linearity maternal age)*

Notes: Figures shows the association between maternal age and birth outcomes. As birth outcomes we measure birth complications as any maternal diagnosis indicating birth complications at birth (ICD 8 and 9 652-659), low birthweight (< 2500g) and preterm (< 37 weeks pregnancy). Estimates show means at maternal age in years with 95% CI. Number of births are shown by histogram. Sample is restricted to children born 1973–1985, the first years from medical birth registry in Sweden.
Source: SIP. Own calculations.

these sources we can identify all twins by using information on an individuals parish of birth, surname as well as exact date of birth. As the data covers the whole universe of the population minus emigrants, we can estimate the effect of being a twin on infant and child mortality, respectively, for the cohorts born 1932 – 1950. Figure B.5 provides the results for this exercise. We indeed find large differences and higher infant mortality in twin births. Early childhood mortality is however less affected. Estimating the corresponding effects for maternal age and sibling spacing on infant and child mortality is not possible as the multigenerational register only starts in 1968 whereby we cannot link deceased infants and children born 1930-1950 to their siblings and parents.

One part of our analysis relates the estimated dementia impact of early-life factors in relation to the corresponding association of education to anchor effect sizes. The noted impact of education in our analysis is on the lower spectrum of the well-researched education dementia association (Maccora et al., 2020). Previous research shows that the education variable from the

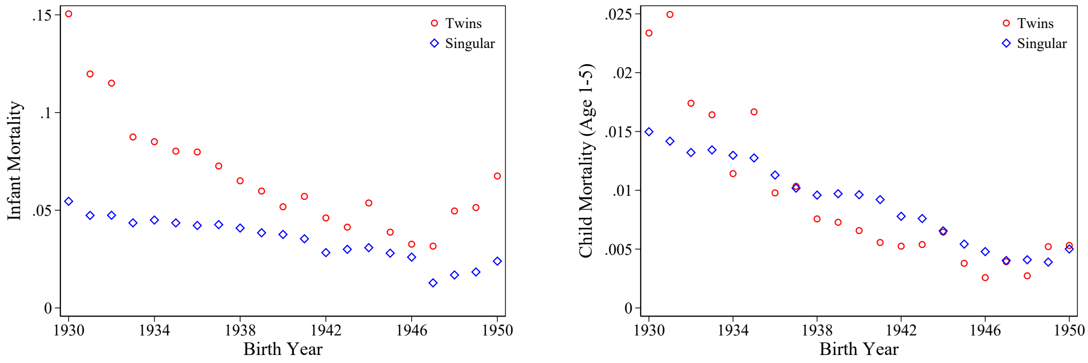

(a) Infant Mortality (Age 0–1) (b) Child Mortality (Age 1–5)

FIGURE B.5 *(Infant and Child Mortality)*

Notes: Figures shows crude infant mortality and child mortality (conditional on survival age 1), separated by twin birth status. Twins are identified by forename, surname, date of birth, parish of birth.
Source: Swedish Death Registry and 1950 Swedish Census. Own calculations.

1970 Census is of high quality (Fischer et al., 2021; Fischer et al., 2022), whereby measurement error in education should not be a severe problem in our application. But measurement error in the dependent variable can lead to an attenuation bias. For binary outcomes, Meyer and Mittag (2017) derive the exact bias for logistic regression and linear probability models. In our case (see Table B.2) logistic regression and linear probability models deliver almost identical results. Together with the fact that we have moderate sensitivity, we can infer from Meyer and Mittag (2017) that our estimates should be downward biased by about 30-40%.

In order to assess if our education estimates are reasonable, we compare our results with findings regarding the association between education and dementia risk using better dementia diagnostics data. Specifically we compare our results to the results in Dekhtyar et al. (2016) based on SNAC-K data. We first re-code our education variable following the definition used in Dekhtyar et al. (2016): elementary schooling (less than 8 years of schooling), secondary education (8-10 years of schooling) and post-secondary and university education (>10 years of schooling) and estimate the education effect with the full population.

The point estimates in Figure B.6 suggest that there is substantial attenuation bias for secondary education, in line with our assumptions. This results further justifies our anchoring approach. If the attenuation bias between education and twin birth association, respectively, is of similar magnitude and with measurement error only present in the dependent variable, bias is proportional for different regressors Meyer and Mittag (2017) and cancels our when used as a ratio. In other words, once, we agree on education is a relevant risk factor, the relative effect of other risk factors can be estimated from our register data.

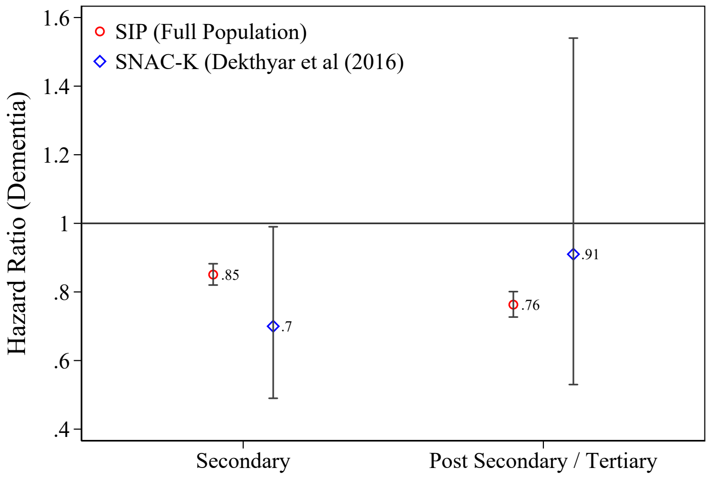

FIGURE B.6 *(Education and Dementia)*: The figure shows estimates for secondary education and post-secondary/tertiary education. SNAC-K estimates are taken from Dekhtyar et al. (2016). Estimates for our sample are restricted to individuals 75 or older, to make sample restrictions comparable.

One of our identified risk exposures is tight birth-spacing. We complement the baseline analysis with an placebo analysis where we restrict the sample to only mothers experiencing at least one short interval birth (which harmonises the sample of families and control for confounding into small interval births) and then estimate the interval effect for the next oldest and next youngest sibling, respectively. Figure B.7 shows the results. First we note that the main effect slightly attenuates, which suggests that part of our main effect is driven by confounding. This is in line with the attenuated effects noted in our within sibling comparison. Second, that effects for the older and younger sibling, are comfortingly zero.

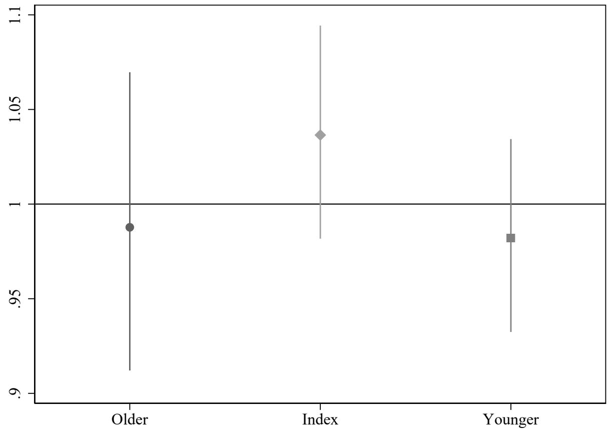

FIGURE B.7 *(Index Spacing)*: The figure shows effects on the index birth along with the older sibling and younger sibling. Sample is restricted to mothers with having at least one short interval birth.

References

Bonsang, E., & Skirbekk, V. (2022). Does childbearing affect cognitive health in later life? evidence from an instrumental variable approach. *Demography*, *59*(3), 975–994.

Callas, P. W., Pastides, H., & Hosmer, D. W. (1998). Empirical comparisons of proportional hazards, poisson, and logistic regression modeling of occupational cohort data. *American journal of industrial medicine*, *33*(1), 33–47.

Dekhtyar, S., Wang, H.-X., Fratiglioni, L., & Herlitz, A. (2016). Childhood school performance, education and occupational complexity: A life-course study of dementia in the kungsholmen project. *International Journal of Epidemiology*, *45*(4), 1207–1215.

Fischer, M., Gerdtham, U.-G., Heckley, G., Karlsson, M., Kjellsson, G., & Nilsson, T. (2021). Education and health: Long-run effects of peers, tracking and years. *Economic Policy*, *36*(105), 3–49.

Fischer, M., Heckley, G., Karlsson, M., & Nilsson, T. (2022). Revisiting sweden's comprehensive school reform: Effects on education and earnings. *Journal of Applied Econometrics*, *37*(4), 811–819.

Koullali, B., Van Zijl, M. D., Kazemier, B. M., Oudijk, M. A., Mol, B. W., Pajkrt, E., & Ravelli, A. C. (2020). The association between parity and spontaneous preterm birth: A population based study. *BMC Pregnancy and Childbirth*, *20*, 1–8.

Maccora, J., Peters, R., & Anstey, K. J. (2020). What does (low) education mean in terms of dementia risk? a systematic review and meta-analysis highlighting inconsistency in measuring and operationalising education. *SSM-Population Health*, *12*, 100654.

Manski, C. F. (1990). Nonparametric bounds on treatment effects. *The American Economic Review*, *80*(2), 319–323.

Meyer, B. D., & Mittag, N. (2017). Misclassification in binary choice models. *Journal of Econometrics*, *200*(2), 295–311.

Read, S. L., & Grundy, E. M. (2017). Fertility history and cognition in later life. *Journals of Gerontology Series B: Psychological Sciences and Social Sciences*, *72*(6), 1021–1031.

Rizzuto, D., Feldman, A. L., Karlsson, I. K., Dahl Aslan, A. K., Gatz, M., & Pedersen, N. L. (2018). Detection of dementia cases in two swedish health registers: A validation study. *Journal of Alzheimer's Disease*, *61*(4), 1301–1310.

Zhang, Y., Fletcher, J., Lu, Q., & Song, J. (2023). Gender differences in the association between parity and cognitive function: Evidence from the uk biobank. *Social Science & Medicine*, 115649.
